# Characterisation of *Salmonella enterica *serotype Typhimurium isolates from wild birds in northern England from 2005 – 2006

**DOI:** 10.1186/1746-6148-4-4

**Published:** 2008-01-29

**Authors:** Laura A Hughes, Sara Shopland, Paul Wigley, Hannah Bradon, A Howard Leatherbarrow, Nicola J Williams, Malcolm Bennett, Elizabeth de Pinna, Becki Lawson, Andrew A Cunningham, Julian Chantrey

**Affiliations:** 1 National Centre for Zoonosis Research, University of Liverpool, Leahurst, Neston, Cheshire, CH64 7TE, UK; 2 Health Protection Agency, Laboratory of Enteric Pathogens, 61 Colindale Avenue, London, NW9 5EQ, UK; 3 Institute of Zoology, Zoological Society of London, Regent's Park, London, NW1 4RY, UK

## Abstract

**Background:**

Several studies have shown that a number of serovars of *Salmonella enterica *may be isolated from wild birds, and it has been suggested that wild birds may play a role in the epidemiology of human and livestock salmonellosis. However, little is known about the relationship between wild bird *S. enterica *strains and human- and livestock- associated strains in the United Kingdom. Given the zoonotic potential of salmonellosis, the main aim of this study was to investigate the molecular epidemiology of *S. enterica *infections in wild birds in the north of England and, in particular, to determine if wild bird isolates were similar to those associated with disease in livestock or humans.

**Results:**

Thirty two *Salmonella enterica *isolates were collected from wild birds in northern England between February 2005 and October 2006, of which 29 were *S. enterica *serovar Typhimurium (*S*. Typhimurium); one *S*. Newport, one *S*. Senftenberg, and one isolate could not be classified by serotyping. Further analysis through phage typing and macro-restriction pulsed-field gel electrophoresis indicated that wild passerine deaths associated with salmonellosis were caused by closely-related *S*. Typhimurium isolates, some of which were clonal. These isolates were susceptible to all antimicrobials tested, capable of invading and persisting within avian macrophage-like HD11 cells *in vitro*, and contained a range of virulence factors associated with both systemic and enteric infections of birds and mammals. However, all the isolates lacked the *sopE *gene associated with some human and livestock disease outbreaks caused by *S*. Typhimurium.

**Conclusion:**

The wild bird isolates of *S. enterica *characterised in this investigation may not represent a large zoonotic risk. Molecular characterisation of isolates suggested that *S*. Typhimurium infection in wild passerines is maintained within wild bird populations and the causative strains may be host-adapted.

## Background

Many studies have shown that a range of *Salmonella enterica *serovars can be isolated from both dead [1-4] and live wild birds [2,5-8] and several studies have suggested that wild birds are important in the epidemiology of human and livestock salmonellosis [9-11]. A number of these studies have used phenotypic methods to characterise isolates, including serotyping, phage typing and antimicrobial sensitivity profiling coupled with epidemiological analysis. These are all useful approaches, but the techniques used to differentiate isolates mean that conclusions that can be drawn from such studies are sometimes limited.

Although there is evidence to suggest that certain strains of *S*. *enterica *subsp. *enterica *serovar Typhimurium (*S*. Typhimurium) are associated with different groups of wild birds [3,4] it is not known if the *Salmonella *strains that cause mortality in UK wild birds, particularly garden birds, are the result of strains belonging to the same clones, a limited range of strains or many different strains, perhaps with differing host and/or geographic ranges. In addition, little is known about the relationship between wild bird *Salmonella *strains and human and livestock-associated strains in the UK, particularly with respect to the virulence genes they contain and their antimicrobial sensitivity profiles. As salmonellosis in wild birds may be zoonotic or transmissible to livestock, it would be of value to understand these relationships in greater detail to assess the risk of wild birds as reservoirs or vectors of *Salmonella *infections.

The main aim of this study was to investigate the molecular epidemiology of *S. enterica *isolates from wild birds in the north of England and, in particular, to determine whether or not the characteristics of 'wild bird' isolates were similar to those of isolates associated with disease in livestock, in particular poultry, or human cases. The relatedness of isolates was determined through a combination of phage typing and macro-restriction pulsed-field gel electrophoresis (PFGE). The presence of a range of virulence-associated genes involved in both avian systemic disease and enteritis in mammals was also determined by PCR (virulotyping). The ability of *Salmonella *strains to invade and survive in host macrophages, an *in vitro *correlate of the ability to cause systemic infection, was determined, and the susceptibility to antimicrobials was determined for each isolate.

## Results

### Bacterial isolates

Thirty two *Salmonella enterica *isolates were collected from wild birds in Northern England from February 2005 until October 2006. *Salmonella *Typhimurium was the most common serotype identified (n = 29), of which the most common definitive bacteriophage type (DT) was DT 56 (n = 23) followed by DT 40 (n = 3), DT 41 (n = 2) and phage type (PT) U277 (n = 1). The *S*. Typhimurium isolates were from 9 greenfinches (*Carduelis chloris*), 8 Eurasian siskins (*Carduelis spinus*), 6 house sparrows (*Passer domesticus*), 2 goldfinches (*Carduelis carduelis*), 2 common starlings (*Sturnus vulgaris*), 1 collared dove (*Streptopelia decaocto*) and 1 wood pigeon (*Columba palumbus*). *S*. Newport (n = 1) and *S*. Senftenberg (n = 1) were isolated from a black-headed gull (*Larus ridibundus*) and a herring gull (*Larus argentatus*) respectively. One further isolate from a herring gull could not be classified by serotyping. Nineteen infected birds were female, 10 were male and the sex of 3 birds was not determined. Twelve birds were juveniles (in their first year), 11 were adults, and nine could not be aged.

The majority of isolates were from dead birds, but three live birds; one house sparrow and two common starlings also yielded *Salmonella *from their faeces. None of these three birds had obvious clinical signs associated with salmonellosis. All isolates were collected between August and April, with a peak of isolations in January (35%) and February (23%): no *Salmonella *was isolated from dead or live birds between May and July (Table 1).

**Table 1:** Characteristics of *Salmonella enterica *isolates and their wild bird hosts. Isolates are listed in order of the location at which *Salmonella *infected birds were found or sampled.

Origin	Sample date	Location	Age	Sex	Dead/alive	Serotype	Phage type	PFGE pattern
House sparrow	02/02/05	1	Unknown	female	Alive	Typhimurium 4,12:i	DT56	5
Herring gull	15/09/05	2	Juvenile	unknown	Dead	I Rough: i: 1,2	Unknown	3
House sparrow	26/10/05	3	Unknown	male	Dead	Typhimurium 4,12:i	DT40	6
Greenfinch	22/10/05	4	Adult	male	Dead	Typhimurium 4,12:i	DT40	6
Greenfinch	19/11/05	5	Adult	male	Dead	Typhimurium 4,12:i	DT56	5
Greenfinch	09/01/06	5	Juvenile	male	Dead	Typhimurium 4,12:i	DT56	5
Eurasian siskin	15/01/06	5	Adult	female	Dead	Typhimurium 4,12:i	DT56	5
Goldfinch	03/01/06	6	Juvenile	unknown	Dead	Typhimurium 4,12:i	DT56	5
House sparrow	29/12/05	7	Juvenile	male	Dead	Typhimurium 4,12:i	PT U277	5
House sparrow	09/01/06	8	Unknown	male	Dead	Typhimurium 4,12:i	DT56	5
Eurasian siskin	20/01/06	9	Unknown	male	Dead	Typhimurium 4,12:i	DT56	5
Eurasian siskin	20/01/06	9	Juvenile	male	Dead	Typhimurium 4,12:i	DT56	5
Eurasian siskin	26/01/06	9	Adult	male	Dead	Typhimurium 4,12:i	DT56	5
Eurasian siskin	06/04/06	9	Adult	female	Dead	Typhimurium 4,12:i	DT56	5
House sparrow	18/01/06	10	Unknown	male	Dead	Typhimurium 4,12:i	DT56	5
Greenfinch	01/02/06	11	Juvenile	male	Dead	Typhimurium 4,12:i	DT56	5
Collared dove	27/01/06	12	Adult	female	Dead	Typhimurium 4,12:i	DT56	5
Wood pigeon	05/02/06	13	Unknown	male	Dead	Typhimurium 4,12:i	DT56	5
House sparrow	19/02/06	13	Unknown	male	Dead	Typhimurium 4,12:i	DT56	5
Eurasian siskin	08/02/06	14	Adult	male	Dead	Typhimurium 4,12:i	DT56	5
Greenfinch	09/02/06	15	Juvenile	female	Dead	Typhimurium 4,12:i	DT56	5
Greenfinch	15/10/06	15	Adult	male	Dead	Typhimurium 4,12:i	DT56	5
Greenfinch	10/03/06	16	Juvenile	female	Dead	Typhimurium 4,12:i	DT56	5
Greenfinch	20/02/06	17	Adult	female	Dead	Typhimurium 4,12:i	DT56	5
Eurasian siskin	14/03/06	18	Juvenile	male	Dead	Typhimurium 4,12:i	DT56	5
Eurasian siskin	23/03/06	19	Juvenile	male	Dead	Typhimurium 4,12:i	DT40	4
Greenfinch	28/04/06	20	Adult	male	Dead	Typhimurium 4,12:i	DT56	5
Common starling	16/08/06	21	Unknown	female	Alive	Typhimurium 4,5,12:i	DT41	3
Common starling	16/08/06	21	Unknown	female	Alive	Typhimurium 4,5,12:i	DT41	3
Goldfinch	17/10/06	22	Adult	male	Dead	Typhimurium 4,12:i	DT56	5
Herring gull	02/08/05	23	Juvenile	female	Dead	Seftenberg	NA	1
Black-headed gull	18/08/05	24	Juvenile	unknown	Dead	Newport	NA	2

### Post-mortem examination

**Figure 1:**
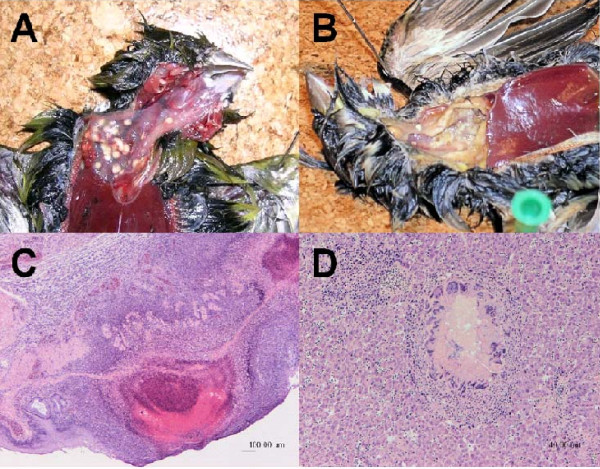
**Gross- and histo-pathology associated with *Salmonella *infection in wild passerine species**. A: Greenfinch crop: multifocal necrotic ingluvitis. B: Greenfinch crop: diffuse necrotic ingluvitis. C: Greenfinch crop (Haematoxylin and Eosin stain): one central and one peripheral nodule of necrotic crop mucosa and submucosa with haemorrhage and surrounded by zones of infiltrating leucocytes. D: Greenfinch liver (Haematoxylin and Eosin stain): multifocal hepatic necrosis surrounded by a zone of macrophages and multinucleate giant cells which is further surrounded by lymphocytes.

Post-mortem examinations were performed on 26 of the 29 dead birds. Twenty three of these birds (88%) were considered to have a poor body condition, as assessed by pectoral muscle mass, and three (12%) had a normal body condition. Seventeen birds (65%) had a multifocal to diffuse, moderate to severe fibrinonecrotic thickening of the crop mucosa (ingluvitis) (Figure 1A,B,C), often with inflammation extending to the underlying connective tissue and muscle. This pathology represented the most frequently reported gross finding. Birds with these crop lesions sometimes also had a fibrinonecrotic hepatitis (Figure 1D) and or splenitis (9 birds, 35%) and several had evidence of moderate to severe, locally extensive small intestinal haemorrhage (6 birds, 23%). Salmonellosis was considered the cause of death in 20 (77%) of the birds examined and was considered an incidental finding in two (8%) birds (a greenfinch and a Eurasian siskin). The role of salmonellosis in the death of four birds was inconclusive from post-mortem examination (a Eurasian siskin, a house sparrow, a wood pigeon and a collared dove); three of these birds were of poor body condition but no lesions typically associated with salmonellosis were found, and one bird was too decomposed to conduct a conclusive post-mortem examination. None of the gull carcases from which *Salmonella *was isolated were subject to a full post-mortem examination.

### Antimicrobial susceptibility testing and PCR-virulotyping

**Figure 2:**
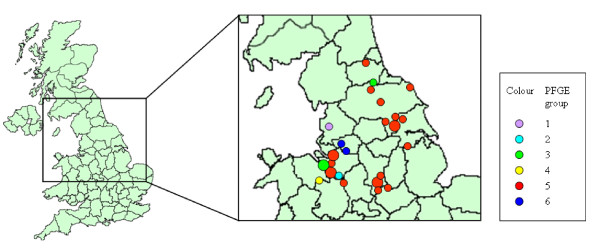
**Map of the distribution of birds from which *Salmonella enterica *were isolated**. Points on the map are colour coded according to the pulsed-field gel electrophoresis banding patterns obtained using *Xba1 *and *Spe1 *enzymes. Larger points represent sites from which more than one *Salmonella *infected dead bird was found.

Each of the isolates were found to be susceptible to all of the antimicrobials tested, and all contained *prgH*, *sopB*, *invA*, *spiC*, *sifA*, *misL*, *pipD*, *iroN*, *sitC*, *orfL *genes as detected by PCR. The fimbrial associated virulence gene, *pefA*, was absent from most isolates apart from three, two of which were *S*. Typhimurium DT 41 isolates from live starlings caught at the same location and date. The other isolate containing *pefA *was the isolate that could not be characterised by serotyping from a dead herring gull, which was sampled at a different location from, and one year later than, the two live starlings. These three isolates also shared the same unique, pulsed field pattern (Figure 2 – green markers). The *sopE *gene, which has been associated with enteritis and epidemics, particularly affecting humans, was absent from all isolates except the control strain *S*. Typhimurium SL1344.

### Pulsed-field gel electrophoresis

**Figure 3:**
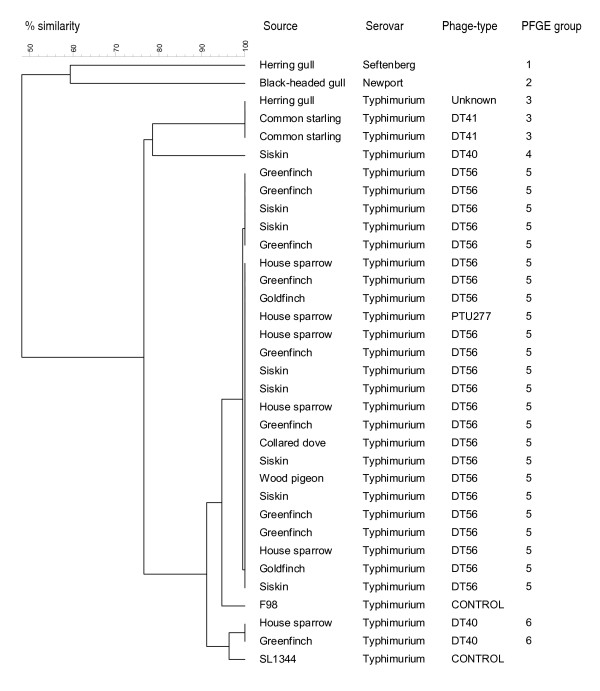
Dendrogram showing the genetic similarity (%) between *Salmonella enterica *isolates digested with *Xba1 *restriction enzyme.

Pulsed-field gel electrophoresis (PFGE) revealed six *Xba*I banding patterns, labelled for the purposes of this analysis as 1–6. Each consisted of 13 or 14 DNA fragments, with relative molecular weights ranging from 48.5 kb to 1018.5 kb (Figure 3). Two patterns (1 and 2) were unique to the single isolates of *S*. Senftenberg and *S*. Newport (respectively). Four patterns were identified amongst the *S*. Typhimurium isolates; two (groups 4 and 6) amongst isolates belonging to the phage type DT 40. Both DT 41 isolates clustered in pulsed-field group 3 together with the isolate that could not be identified by serotyping, and DT 56 isolates all clustered in group 5. The *S*. Typhimurium PT U277 isolate also had a group 5 banding pattern. Very high genetic similarity (>90%) was seen within each pulsed-field group. Across all *S*. Typhimurium isolates, analysis of the PFGE banding patterns suggested an overall genetic similarity of 77%, with a genetic similarity of 99% among DT 56 and PT U277 isolates. These *Xba*1 PFGE groups were confirmed by PFGE after digestion with the *Spe*1 restriction enzyme.

Multiple birds were examined from three of 24 locations (Figure 2, Table 1) (<3 month interval between cases at same site). The same phage type and PFGE profile was found in all isolates cultured from the same location.

### Cell invasion assays

**Figure 4:**
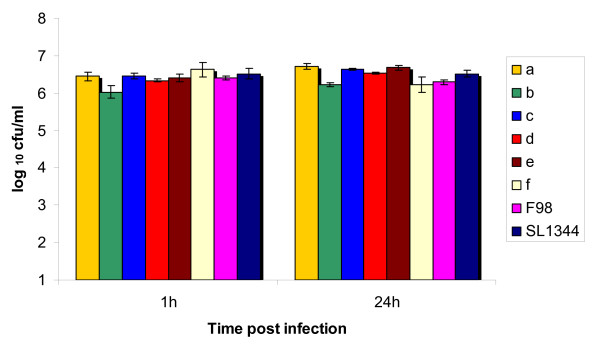
**Uptake and persistence of *Salmonella *isolates from wild birds in avian HD11 cells**. Viable counts of a sub-sample of *Salmonella enterica *serovar Typhimurium isolates, plus an isolate that could not be characterised by serotyping, expressed as CFU/ml were determined on nutrient agar after the ability of the isolates to invade and persist in avian HD11 cells was assessed using a gentamicin protection assay. Within the sub-sample there were isolates of each phage type and *S*. Typhimurium pulsed field group identified in this study (a – f) plus two control strains (F98 and SL1344).

All *Salmonella *isolates tested showed similar levels of invasiveness for avian HD11 cells, as demonstrated by the bacterial counts performed one hour after infection (Figure 4). In addition, all isolates tested persisted within the cells for 24 hours and in most cases, underwent replication.

## Discussion

The data presented here show that salmonellosis of wild, particularly passerine birds, in the north of England during 2005 and 2006 was caused mainly by a narrow range of possibly host-adapted *S*. Typhimurium strains. These strains are generally susceptible to antimicrobials in contrast to many human and livestock-associated strains [12,13] such as *S*. Typhimurium DT 104 [12-14]. They contain a range of genes associated with systemic and enteric disease in birds and mammals, but lack *sopE*, a gene that has been associated with some epidemic strains of *S*. Typhimurium in both humans and food animals. The *sopE *gene has been identified in six of 15 outbreak-associated *S*. Typhimurium phage types, but not in the epidemic multiresistant phage type DT 104, which has been the most common *S*. Typhimurium phage type in cases of human infection in England and Wales since 1990 [14].

The majority of *Salmonella *isolates obtained during this study were of serovar Typhimurium, of which four phage types were identified; DT 40, DT 41, DT 56 and PT U277, with DT 56 being the predominant phage type, widespread spatially and temporally over the north of England (Figure 2). As far as we know, *S*. Typhimurium DT 56 has not been reported previously in wild birds from outside of the UK, unlike phage types DT 40 and PT U277 that have been reported in wild birds from Scandinavia [15]. It is possible that the predomination of phage type DT 56 may result from the fact that sample collection was geographically and temporally limited to the north of England in 2005 and 2006, therefore a single clone may have been the origin of most of the DT 56 isolates. This hypothesis would be supported by the results of the PFGE analysis carried out by this study where all DT 56 isolates showed 99% similarity to each other. The majority of *S*. Typhimurium DT 56 and DT 40 isolates were from members of the *Fringillidae *(greenfinch, goldfinch and siskin) and *Passeridae *(house sparrow) families, which is consistent with the findings in previous studies [3,4,16]. In addition, *S*. Typhimurium DT56 was isolated from a wood pigeon and a collared dove. *S*. Typhimurium DT 41 was isolated from starlings and one herring gull: the findings of this study and others would suggest that DT 41 causes only sporadic mortality in 'garden birds' and is more frequently associated with gull and wildfowl species [4].

*S*. Typhimurium DTs 40 and 56 were those most often associated with garden bird mortality, as found in previous studies [4,17,18]. Pulsed-field gel electrophoresis demonstrated that the *S*. Typhimurium isolates in this study belonged to a small number of closely related, sometimes clonal, strains. This suggests that *S*. Typhimurium infection in garden birds is maintained within the wild bird population rather than there being repeated infection of wild birds from other sources. However, we cannot state that transmission never occurs between wild birds and livestock or humans, and wild birds should therefore still be treated as a potential source of *Salmonella *infection. Indeed, a number of studies carried out in Norway and New Zealand have shown an epidemiological link between wild passerines and human *Salmonella *outbreaks [11,19].

The *S*. Typhimurium isolates clustered into four main PFGE groups which, with three exceptions, were closely correlated to phage-type. An isolate of PT U277 showed 99% similarity to the DT 56 isolates that clustered together in PFGE group 5. PFGE analysis grouped 3 DT 40 isolates into 2 patterns (groups 4 and 6) that were separated geographically (Figure 2). Two DT 41 isolates from live starlings sampled at the same location on the same date shared an identical PFGE pattern with the isolate from a dead herring gull that could not be classified by serotyping, sampled at a different location on a different date. Interestingly, these last three isolates were the only ones to contain the fimbrial associated virulence gene *pefA*.

PFGE group 5 contained the largest number of isolates, including all of the DT56 isolates obtained during this study, demonstrating that these isolates are members of a clonal strain. Alley *et al *[19] reported a similar finding that all DT160 isolates examined by PFGE during an outbreak of salmonellosis in wild passerines and humans in New Zealand were indistinguishable and therefore members of a clonal strain. Due to the small number of isolates phage-typed as DT40 and DT41 in this study, it is not possible to comment on their clonality. *S*. Typhimurium DT 40 is known to affect wild birds, particularly passerine species [4], therefore it is important to investigate variation within this phage type further. Similar work carried out on a national scale over a longer time period would be valuable.

Every isolate was found to possess all of the virulence genes that were screened for apart from *pefA *and *sopE*. The *pefA *gene is located on a virulence plasmid rather than the bacterial chromosome [20]. Such plasmids can be serovar-specific, but it has been found that not all isolates of plasmid-bearing serovars contain these plasmids [21,22]. This may explain the low prevalence of this gene among the *Salmonella *isolates obtained in the present study. The rest of the virulence-associated genes screened for are located on *Salmonella *pathogenicity islands (PAIs) 1–5 [21,23,24], and are known to be associated with adhesion, cell invasion and intra-cellular survival. These genes, particularly those associated with the *Salmonella *pathogenicity island 1 (SPI-1) and *Salmonella *pathogenicity island 2 (SPI-2) type III secretion systems, have been well-characterised for their role in both enteritis and systemic infection in mammalian models. Their roles in avian infection are less clear, though the SPI2 system is a requirement for infection and disease in poultry with the avian specific serovars *S*. Gallinarum and *S*. Pullorum [25,26]. Recently SPI1 and SPI2 have been shown to be involved in both systemic and gastrointestinal tract infection of the chicken by *S*. Typhimurium. The possession of these virulence factors would suggest that the isolates investigated in the current study have the ability to cause systemic and enteric salmonellosis in their hosts. In addition, the ability of all strains tested to invade and persist in avian macrophage-like HD11 cells, would also suggest that they have the potential to cause systemic infection in avian species: mutant strains of *Salmonella *incapable of surviving within macrophages *in vitro *are attenuated for systemic virulence [25,27].

Interestingly, the *Salmonella *isolate obtained from a live house sparrow was of the same virulence genotype as the isolates from dead house sparrows and other species. Skyberg *et al *[21] found that a number of virulence genes could be detected in both healthy and sick poultry, indicating that some virulence genes and associated PAIs may be widespread in salmonellae isolated from both healthy and sick birds. It may be that other factors, such as nutritional, environmental or physiological stressors are also involved in the development of clinical disease and mortality, or it could be that these healthy birds had only very recently become infected and had not had time to develop disease. Experimental studies have shown that gastrointestinal carriage of *Salmonella *occurs in passerines after infection; therefore it is possible that healthy birds could be persistent carriers [28].

None of the isolates examined possessed the *sopE *gene, which has been found to be present in some strains of *S*. Typhimurium associated with epidemic disease in both humans and food animals [29] but not with *S*. Typhimurium DT 104 [24]. This may indicate that wild passerine strains of *S*. Typhimurium are generally not involved in the epidemiology of *S*. Typhimurium infection in humans or livestock in the UK. The absence of antibiotic resistance detected in this study would also support this hypothesis, as many *S*. Typhimurium isolates from human or production animal sources are resistant to at least one antibiotic [12-14]. It is possible that the *S*. Typhimurium strains affecting wild passerines are adapted to, and maintained within, the wild bird population. However, this study examined isolates collected only during 2005 and 2006. It would require a more spatially extensive study carried out over a longer time period to gather sufficient evidence to fully support this hypothesis.

The gross post-mortem findings in 20 out of 26 birds examined were similar to those described in previous studies [4,30-33]. Lesions were most commonly present in the crop of the bird, and it has been suggested that ingluvitis and oesophagitis in passerines with salmonellosis indicates that the crop and oesophagus are predilection sites for bacterial invasion. It is possible that progression of this infection to systemic salmonellosis involving other organs (liver and spleen) may occur if the bird's immune system became further compromised [34]. No gross lesions were noted in two dead birds from which *Salmonella *was isolated, a greenfinch and a Eurasian siskin. In these cases where *Salmonella *appeared as an incidental finding, it is impossible to know if these birds would have succumbed to salmonellosis in the future (had they not been killed by some other cause) or for how long the bird had been infected before death. Studies have shown that chickens can carry *Salmonella enterica *containing virulence genes asymptomatically [21], and this must therefore be a possibility in wild birds also. The only way to determine if this is the case would be through a longitudinal study of individual wild birds.

## Conclusion

Molecular characterisation of the *Salmonella *isolates collected from both live and dead wild birds in this study has indicated that wild passerine deaths in northern England associated with salmonellosis during 2005 and 2006 were caused by a small number of closely-related *S*. Typhimurium strains, some of which appear to be clonal. These strains were typically susceptible to antimicrobials, capable of invading and persisting within avian macrophage-like cells and contained a range of virulence factors associated with intra-cellular survival, adhesion and invasiveness. All the isolates tested lacked the *sopE *gene associated with some *S*. Typhimurium disease outbreaks in humans and livestock, which suggests that it is unlikely that these isolates represent a large zoonotic risk. These findings also suggest that *S*. Typhimurium infection in wild passerines is maintained within wild bird populations and may be host-adapted. To investigate this further, similar work would need to be carried out on a national scale over a longer time period.

## Methods

### Bacterial isolates

Thirty two *Salmonella *isolates were collected from wild birds from a total of 24 sites across northern England (Table 1 and Figure 2) between July 2004 and January 2007. The number of isolates collected from each location is detailed in Table 1. Isolates were obtained from a total of 2100 faecal samples collected from apparently healthy wild birds caught primarily for ringing (85.5% of samples), dead birds submitted to a local wildlife hospital (8.5% of samples), and dead birds collected from members of the public through the Garden Bird Health Initiative (6% of samples) [35]. Where possible, a full post-mortem examination was carried out on dead birds.

All faecal samples collected were processed in the laboratory by the author within 24 hours of sample collection. *Salmonella *bacteria were isolated using a routine method [36]: briefly faecal samples were enriched aerobically in peptone buffer at 37°C for 24 hours followed by incubation in Rappaport-Vassiliadis broth at 42°C in aerobic conditions again for 24 hours, before inoculation onto Rappaport-Vassiliadis agar and incubation for 24 hours at 37°C. Colonies suspected of being salmonella were confirmed using an API20E kit (Biomérieux, l'Etoile, France) according to the manufacturer's instructions. Isolates were serotyped by performing slide agglutination tests with *Salmonella *O and H group antisera (Pro-Lab, Neston, UK). All *S*. Typhimurium isolates were phage-typed as previously described [37,38].

### Antimicrobial susceptibility testing

*Salmonella *Typhimurium isolates were tested for antimicrobial susceptibility by disk diffusion on iso-sensitest agar (LabM, Bury, UK) using British Society for Antimicrobial Chemotherapy (BSAC) guidelines for testing enterobacteriaceae [39]. Isolates were tested for susceptibility to amikacin (AMK), amoxicillin/clavulanic acid (AMX), ampicillin (AMP), cefpodoxime (CPD), cefoxitin (FOX), chloramphenicol (CHL), ciprofloxacin (CIP), nalidixic acid (NAL), oxytetracycline (OTC) and trimethoprim/sulfamethoxazole (SXT).

### PCR virulotyping

Crude DNA extracts were prepared for each isolate by boiling in water for 20 minutes, and screened by polymerase chain reaction (PCR) for the presence of several genes thought to be associated with virulence [21,23,24] (Table 2). The reaction mixtures and cycling conditions were the same for all reactions: 1.25 units Taq DNA polymerase (ABgene, Epsom, UK); 75 mM Tris-HCl (pH 8.8 at 25°C); 20 mM (NH_4_)_2_SO_4_; 2.5 mM MgCl_2_; 0.01% (v/v) Tween 20; 0.2 mM each of dATP, dCTP, dGTP and dTTP; 4 mM forward and reverse primers; with 1 microlitre of the DNA template in a final reaction volume of 25 μl. DNA amplification was carried out in a Thermo Hybaid thermocycler (ABgene, Epsom, UK) with an initial denaturation step at 94°C for 3 minutes followed by 30 cycles of amplification (denaturation at 94°C for 1 minute, annealing at 55°C for 1 minute and extension at 72°C for 1 minute) with a final extension step at 72°C for 5 minutes, followed by a holding temperature of 10°C. The PCR products were separated by gel electrophoresis in a 2% agarose gel in Tris-acteate buffer. Ethidium bromide was added to the agarose, and the gel was visualized under ultra-violet light. Amplicon size was determined by comparison with ΦX174 Hae III Digest DNA marker (ABgene, Epsom, UK). Two control *S*. Typhimurium strains were used: *S*. Typhimurium SL1344 and *S*. Typhimurium F98 [40,41].

**Table 2:** Virulence genes and PCR primers used to test screen *Salmonella *isolates.

**Virulence gene**	**Pathogenicity island**	**Gene function**	**Broad action**	**Primer sequence (5' to 3')**
*prgH*	SPI-1	Type III secretion system apparatus	Invasion of macrophages	F: GCCCGAGCAGCCTGAGAAGTTAGAAA R: TGAAATGAGCGCCCCTTGAGCCAGTC
*sopB*	SPI-1	Type III secreted effector protein	Invasion of macrophages	F: CGGACCGCCCAGCAACAAAACAAGAAGAAG R: TAGTGATGCCCGTTATGCGTCAGTGTATT
*sopE*	SPI-1	Type III secreted effector protein	Invasion of macrophages	F: TCAGTTGGAATTGCTGTGGA R: TCCAAAAACAGGAAACCACAC
*invA*	SPI-1	Type III secretion system apparatus	Invasion of macrophages	F: CTGGCGGTGGGTTTTGTTGTCTTCTCTATT R: AGTTTCTCCCCCTCTTCATGCGTTACCC
*sitC*	SPI-1	Iron transport	Invasion of macrophages/iron acquisition	F: CAGTATATGCTCAACGCGATGTGGGTCTCC R: CGGGGCGAAAATAAAGGCTGTGATGAAC
*spiC*	SPI-2	Type III secretion system	Survival in macrophages	F: CCTGGATAATGACTATTGAT R: AGTTTATGGTGATTGCGTAT
*sifA*	SPI-2	Type III secreted effector protein	Survival in macrophages	F: TTTGCCGAACGCGCCCCCACACG R: GTTGCCTTTTCTTGCGCTTTCCACCCATCT
*misL*	SPI-3	Involved in intramacrophage survival	Survival in macrophages	F: GTCGGCGAATGCCGCGAATA R: GCGCTGTTAACGCTAATAGT
*orfL*	SPI-4	Adhesin/autotransporter	Survival in macrophages/colonisation	F: GGAGTATCGATAAAGATGTT R: GCGCGTAACGTCAGAATCAA
*pipD*	SPI-5	Type III secreted effector-associated with SPI-1 system	Enteritis	F: CGGCGATTCATGACTTTGAT R: CGTTATCATTCGGATCGTAA
*iroN*	NA	Siderophore (iron acquisition)	Associated with iron usage	F: ACTGGCACGGCTCGCTGTCGCTCTAT R: CGCTTTACCGCCGTTCTGCCACTGC
*pefA*	NA	Fimbriae	Movement	F: GCGCCGCTCAGCCGAACCAG R: CAGCAGAAGCCCAGGAAACAGTG

### Pulsed-field gel electrophoresis (PFGE)

Block preparation and PFGE were performed according to the Standardised PulseNet Rapid *E. coli *PFGE method with slight modifications [42]. Genomic DNA from *Salmonella *isolates was digested using 50 U per sample *XbaI *(Promega, Southampton, UK) or 15 U per sample *SpeI *(Promega) enzymes for 2 hours or 22 hours respectively. Macro-restriction digested fragments were separated on a 1% agarose gel (pulsed-field certified, BioRad Laboratories, Hertfordshire, UK), at 210 volts for 19 hours at 14°C on a CHEF DRIII system (Bio-Rad Laboratories). Pulse times were ramped from 2.2–54.2 seconds and a reorientation angle of 120° was applied. Bacteriophage λ DNA concatemers (Bio-Laboratory) embedded in 1% LMP agarose were used as molecular weight markers, and *S*. Typhimurium SL1344 and *S*. Typhimurium F98 were again used as controls. Gels were stained for 20 minutes with 1% ethidium bromide solution and visualised using UV light. BioNumerics version 4.0 (Applied Maths BVBA, Sint-Martens-Latem, Belgium) software was used for image analysis. A percentage similarity between pulse-field banding patterns was computed according to the Dice similarity coefficient method with a 2% tolerance window, and a dendrogram was constructed using the UPGMA (unweighted pair group method with averages).

### Cell invasion assays

Six isolates of *S*. Typhimurium, including isolates from both live and dead birds plus a representative sample of each *S*. Typhimurium PFGE pattern and phage type identified, were tested for their ability to invade and persist in avian macrophage-like HD11 cells using a gentamicin protection assay as previously described by Jones *et al *[25]. Chicken macrophage-like cell line HD11 was used for the assays [43]. For bacterial counts, a modified Miles-Misra method was used to estimate the number of bacteria per millilitre of macrophage cells one hour post-infection and 24 hours post-infection. Each assay was repeated 3 times for each bacterial isolate and an average bacterial count is presented in the results.

## Authors' contributions

LH performed post-mortem examinations and *Salmonella *isolations. LH and SS conducted the majority of the experimental work and co-wrote the manuscript. PW designed the virulotyping, PFGE and cell invasion experiments, helped design the study and co-wrote the manuscript. HB conducted the initial pilot study. AHW helped conduct and analysed the PFGE data. NW designed and helped conduct the antimicrobial sensitivity testing. MB helped design and analyse the study and co-wrote the manuscript. EDP performed the phage typing and contributed to the writing of the manuscript. BL and AC coordinated the Garden Bird Health Initiative through which a number of the samples were collected, and contributed towards the study design. JC performed post-mortem examinations and their analysis, helped in the study design and co-wrote the manuscript. All authors read and approved the final manuscript.
